# Response of Bacteria Community to Long-Term Inorganic Nitrogen Application in Mulberry Field Soil

**DOI:** 10.1371/journal.pone.0168152

**Published:** 2016-12-15

**Authors:** Cui Yu, Xingming Hu, Wen Deng, Yong Li, Guangming Han, Chao Xiong

**Affiliations:** Industrial Crops Institute of Hubei Academy of Agricultural Sciences, Wuhan, China; National Renewable Energy Laboratory, UNITED STATES

## Abstract

The bacterial community and diversity in mulberry field soils with different application ages of inorganic nitrogen fertilizer (4Y, 4-year-old; 17Y, 17-year-old; 32Y, 32-year- old) were investigated using next-generation sequencing. The results demonstrated that the application ages of nitrogen fertilizer significantly altered soil bacterial community and diversity. Soil bacterial Shannon diversity index and Chao 1 index decreased with the consecutive application of nitrogen fertilizer, and the 4Y soil exhibited the highest bacterial relative abundance and diversity. Of 45 bacterial genera (relative abundance ratio of genera greater than 0.3%), 18 were significantly affected by the plant age, and seven belong to *Acidobacteria*. The relative abundances of *Acidobacteria* Gp 1, Gp4 and Gp6 in the 4Y soil were significantly lower than that of in the 17Y and 32Y soils. However, the relative abundance of *Pseudononas* sp. in the 4Y soil was significantly higher than that of in the 17Y and 32Y soils. Most microbial parameters were significantly affected by soil pH and organic matter content which were significantly changed by long-term application of inorganic nitrogen fertilizer.

## Introduction

Soil microorganisms play critical roles in nutrient transformation and soil fertility maintenance [1–3]. It has been reported that soil microbial community composition and diversity are affected by nitrogen fertilizer management [4–11]. The influences of nitrogen fertilizer application on soil microbial community is very complex and may be related to nitrogen application patterns (application rates and durations), soil biotic and abiotic factors [10–13]. Previous studies have shown that long-term nitrogen application reduces microbial diversity [8, 14]. Another set of studies suggested that a certain amount of nitrogen has no effect on the soil microbial community composition or diversity [5–7]. However, the effects of the application of inorganic nitrogen fertilizer on soil microbial community composition and diversity are not well understood in mulberry plantations.

Mulberry (*Morus* spp.) leaves are the sole food of silkworms (*Bombyx mori*). However, the leaf yields of mulberry in Hubei Province which is one of the main silk-producing regions in China are about half lower than those in other leading silk-producing provinces, such as Guangxi, Guangdong and Zhejiang [15]. A previous survey suggested that fertilizer application has become an important factor that limits leaf yield and quality in Hubei province [15]. In order to obtain large quantities of leaves, farmers often use inorganic fertilizers, particularly nitrogen fertilizers. Currently, nitrogen fertilizers are applied to mulberry plantations at the high rate of 454 kg N ha^–1^ y^–1^ in Hubei Province [15]. However, few studies have investigated the effects of this high input level on the changes in soil microbial community composition and diversity [16–17]. Our objectives in this study were to understand the changes in bacterial community composition and diversity when only nitrogen fertilizers were applied over a longer period and to provide a theoretical basis for fertilization management on mulberry plantations.

## Material and Methods

### Site description and experimental design

The experimental site was established at the experimental farm of the Industrial Crops Institute at the Hubei Academy of Agricultural Sciences in Hubei Province, China (30°35’N, 114°37’E, 50 m a.s.l.). This region has a typical subtropical monsoon climate with an average annual precipitation of 1, 269 mm and an average temperature between 15.8°C and 17.5°C.

To assess the effects of the application of nitrogen fertilizer on soil microbial properties, a completely randomized block design with three replicates was designed. The mulberry fields were constructed on wasteland, and Husang 32 mulberry (*Morus alba* L.) was planted in 2010, 1997, and 1982 with a row × line spacing of 1.0 m × 1.5 m. The area of each plot was 66.7 m^2^. The soils are clay loam. After the mulberry was planted, only urea nitrogen fertilizer was applied at an application rate of 450 kg N ha^–1^; thus, the duration of nitrogen application were 4, 17, and 32 years old, respectively, when the soil samples were collected. The fertilizers were applied twice each year, with 40% in the March and 60% after pruning in June. The physico-chemical properties of the soil in November 2013 and 2014 are shown in S1 Table. The soil pH was measured by preparing a 1:2.5 slurry of fresh soil to water (v/v) and using a pH meter (OHAUS, Starter 3C). The soil organic matter was determined using the standard Walkley-Black potassium dichromate oxidation method [18]. The available nitrogen content was measured using the alkali-hydrolysis and diffusion method. The available phosphorus content was extracted using 0.5 M NaHCO_3_ and measured with the Olsen method [19]. The available potassium was extracted in 1 M NH_4_OAc (1:10 soil: solution ratio) for 1 h and analyzed using atomic absorption spectrophotometry [20].

### Sample collection and preparation

Fifteen soil cores (50 cm from the mulberry tree trunk at a depth of 0–20 cm) (using GPS to fix the position) were periodically collected at random from per plot of nitrogen fertilizer application of 4-year-old (4Y), 17-year-old (17Y) and 32-year-old (32Y) and mixed to form one composite sample, respectively. The samplings took place in November 2013 and 2014. The samples were transported in a car refrigerator to the lab measured immediately within one week after collection. Part of the soil was stored at -80°C for the soil microbiological high-throughput sequencing analysis, and another part of the soil was air-dried, ground and passed through 1mm and 2 mm mesh sieves for the chemical analysis.

### DNA extraction and PCR amplification 16S rRNA

The genome DNA was directly extracted from the soil of November sampling in 2013 and 2014 by using an E.Z.N.A.^®^ Soil DNA kit (Omega Bio-Tec, Inc., USA) according to the manufacturer’s instructions. The quality of the extracted DNA was ensured using 1% agarose gels. The V3–V4 hypervariable regions of 16S rRNA were PCR amplified from the microbial genome DNA by using barcoded fusion primers (forward primers: 341F CCTACACGACGCTCTTCCGATCTN (barcode) CCTACGGGNGGCWGCAG, reverse primers: 805R GACTGGAGTTCCTTGGCACCCGAGAATTCCAGACTACHVGGGTATCTAATCC). The reaction mixtures (50 μl) contained 5 μl of 10× PCR reaction buffer (TakaRa, Japan), 10 ng of DNA template, 0.5 μl of each primer, 0.5 μl dNTPs, and 0.5 μl of plantium Taq DNA polymerase (TakaRa, Japan). The PCR conditions were: 94°C for 3 min, 94°C for 30 sec, annealing at 45°C for 20 s and 65°C for 30 s, repeated for 5 cycles, followed by 94°C for 20 s, 55°C for 20 s, 72°C for 30 s, repeated for 20 cycles, and a final elongation at 72°C for 5 min. The PCR product was excised from a 1.5% agarose gel and purified using a QIAquick Gel Extraction Kit.

### Amplicon sequence and sequence data processing

Barcoded V3 and V4 amplicons were sequenced using the pair-end method by Illumina Miseq (Illumina, San Diego, CA, USA) with a 6-cycle index. Sequences with an average phred score of less than 25 that contain ambiguous bases, a homopolymer run exceeding 6, mismatches in primers or a sequence length of less than 100 bp were removed using the Prinseq software (PRINSEQ-lite 0.19.5). For the V3 and V4 pair-end reads, only the sequences that overlapped by more than 10 bp without any mismatch were assembled according to their overlapping sequences using the Flash software (FLASH v1.2.7). Reads that could not be assembled were discarded. Barcode and sequencing primers were trimmed from the assembled sequences (V3 and V4).

Sequences were clustered and assigned to operational taxonomic units (OTUs) at a 3% dissimilarity level using the Uclust software (uclust v1.1.579). Taxonomic ranks were assigned to each sequence using the Ribosomal Database Project (RDP) Naïve Bayesian Classifier v.2.2 trained on the Greengenes database (Oct, 2012 version) [21]. The abundance count at the genus level was log2 transformed and then normalized as follows: from each log transformed measure, the arithmetic mean of all transformed values was subtracted, and the difference was divided by the standard deviation of all log-transformed values for a given sample. After this procedure, the abundance profiles for all samples exhibited a mean of 0 and a standard deviation of 1. The bacterial diversity is shown by the number of OTUs. Principal Coordinates Analysis (PCoA) in genus level was performed using R (vegan).

## Statistical analysis

The results were analyzed using the SPSS software program (version 10.0 for Windows, Chicago, IL, USA). The differences in the abundance of the individual OTUs and treatment means among the plant age were tested by one-way ANOVA, and significant differences between the means were determined using the Fisher’s least significant difference (LSD) test. Normal distribution and homogeneity of variance were verified by Bartlett’s and Dunnett’ tests. The differences were considered statistically significant when *P* < 0.05. T-tests were performed by Microsoft excel and all p-values were adjusted by False Discovery Rate (FDR) using the BH method by mt.rawp2adjp function in R. To determine the key factor(s) affecting microbial parameters, stepwise multiple regression analysis was applied using the probability criteria of *P* < 0.05 to accept and *P* > 0.1 to remove a variable from the analysis. Pearson correlation analysis was used to test the correlations between the AOA and AOB abundances, Shannon diversity index and the physicochemical parameters.

## Results

### Sequence data and bacterial taxonomic richness

A total of 975,146 paired-end 250-bp reads were acquired from all 18 samples, with 321,344, 344,612 and 309,190 raw reads at the 4Y, 17Y and 32Y soils, respectively (S2 Table). After initial quality control, 931,440 high quality sequences were obtained. On an average, 51,747 sequences were obtained per sample. Based on 97% species similarity, 8,522, 7,067 and 6,844 operational taxonomic units (OTUs) were obtained from samples of 4Y, 17Y and 32Y soils, respectively (S2 Table). The average length of the sequence reads was 440 bp, and they were classified into different taxonomies using uclust [22].

**Fig 1:**
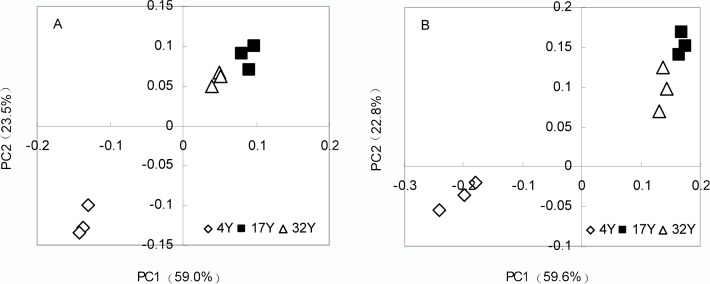
PCoA of unweighted UniFrac distances for the soil microbiota at the nitrogen application of 4-year-old (4Y), 17-year-old (17Y), and 32-year-old (32Y) mulberry field soils in 2013 (A) and 2014 (B).

Both the abundance and diversity index of bacteria in the mulberry field soil were significantly affected by the nitrogen application ages (Table 1). From the numbers of OTUs in the three groups, we found that bacterial diversity decreased as the nitrogen application age increased (Table 1). A stepwise regression analysis revealed that the number of OTUs was correlated with the soil organic matter content and pH (S3 Table). The bacterial relative abundance is reflected in the Chao1 index; the Chao1 index was significantly decreased with the consecutive application of nitrogen fertilizer (*P* < 0.05; Table 1), indicating that the bacterial relative abundance decreased with the nitrogen application ages. The principal coordinates analysis (PCoA) of UniFrac distance matrices indicated that the variation was primarily explained by the application ages of nitrogen. The 4Y soil microbiota clustered separately from the microbiota of the 17Y and 32Y soil along principal coordinate 1 (Fig 1), suggesting that the application ages of nitrogen fertilizer influenced the community structure of the soil bacteria.

**Table 1:** Alpha index of mulberry field soils with the nitrogen application of 4-year-old (4Y), 17-year-old (17Y), and 32-year-old (32Y).

Plant ages		OTU number	Shannon index	Chao1 index
2013	4Y	9605±136.8^a^	7.70±0.11^a^	21256±172.6^a^
	17Y	8328±135.3^b^	7.02±0.06^b^	16733±103.4^b^
	32Y	5102±47.6^c^	5.98±0.26^c^	10397±98.0^c^
2014	4Y	10030±148.2^a^	7.35±0.09^a^	21666±159.8^a^
	17Y	8469±125.3^b^	6.41±0.65^b^	16873±123.7^b^
	32Y	6082±112.1^c^	6.22±0.98^b^	13612±113.5^c^

The data are expressed as the means ± SD (*n* = 3). The superscript letters that differ within a column indicate significant differences between treatments (*P* < 0.05).

### Bacterial community composition in the soil

**Fig 2:**
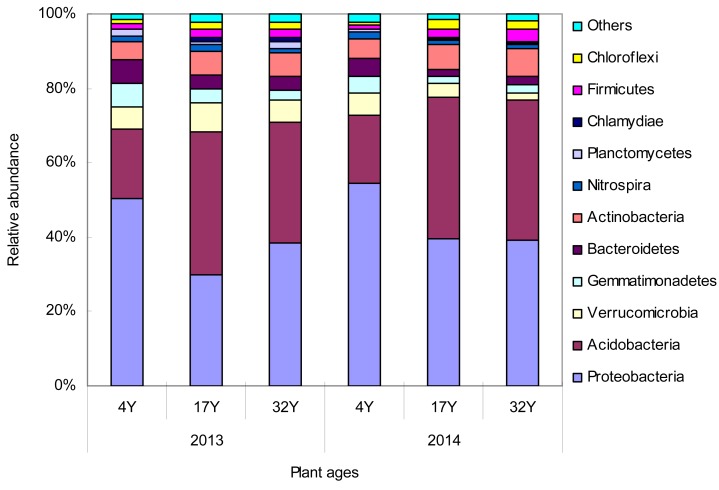
Relative abundance of the dominant Phyla in the nitrogen application of 4-year-old (4Y), 17-year-old (17Y), and 32-year-old (32Y) mulberry field soils. Relative abundances are based on the proportional frequencies of those DNA sequences that could be classified.

At the phylum level, a total of 27 phyla were shared by the three soil samples. The main phyla were as follows: *Proteobacteria*, *Acidobacteria*, *Verrucomicrobia*, *Gemmatimonadetes*, *Bacteroidetes* and *Actinobacteria* (Fig 2). Six phyla (*Proteobacteria*, *Acidobacteria*, *Nitrospira*, *Firmicutes*, *Chloroflexi* and *Bacteroidetes*) differed (*P* < 0.05) among different soils (Table 2). *Proteobacteria* was the most dominant phylum, regardless of the different samples, and comprised 30.0%-54.3% of the total sequences. *Acidobacteria* was the second largest phylum, comprising approximately 18.5%-38.3%. The relative abundance of *Proteobacteria* in the 4Y soil was significantly higher than that of in the 17Y and 32Y soils (*P* < 0.05; Fig 2) and significantly positively correlated with the pH (r = 0.762, *P* < 0.05) and soil organic matter content (r = 0.648, *P* < 0.01) (S4 Table). However, the relative abundances of *Acidobacteria*, *Firmicutes* and *Chloroflexi* in the 4Y soil were significantly lower than that of in the 17Y and 32Y soils (*P* < 0.05; Fig 2). The relative abundance of *Acidobacteria* significantly negatively correlated with the pH (r = –0.663, *P* < 0.05) and soil organic matter content (r = –0.617, *P* < 0.05) (S4 Table).

**Table 2:** Comparisons (T-test) for phylum abundance between the nitrogen application of 4-year-old (4Y), 17-year-old (17Y), and 32-year-old (32Y) mulberry field soils.

**Phyla**	**Relative fold change**	*P* value (* *P* <0.05, ** *P* <0.01)
	**4Y / 17Y**	
*Proteobacteria*	1.70	0.021*
*Acidobacteria*	-2.29	0.010**
*Nitrospira*	1.34	0.032*
*Firmicutes*	-2.65	0.014*
*Chloroflexi*	-2.41	0.016*
*Bacteroidetes*	1.63	0.038*
**Phyla**	**Relative fold change**	*P* value (* *P* <0.05, ** *P* <0.01)
	**4Y / 32Y**	
*Proteobacteria*	1.57	0.032*
*Acidobacteria*	-2.31	0.004**
*Nitrospira*	1.24	0.041*
*Firmicutes*	-2.31	0.024*
*Chloroflexi*	-2.19	0.018*
*Bacteroidetes*	1.37	0.043*

**Fig 3:**
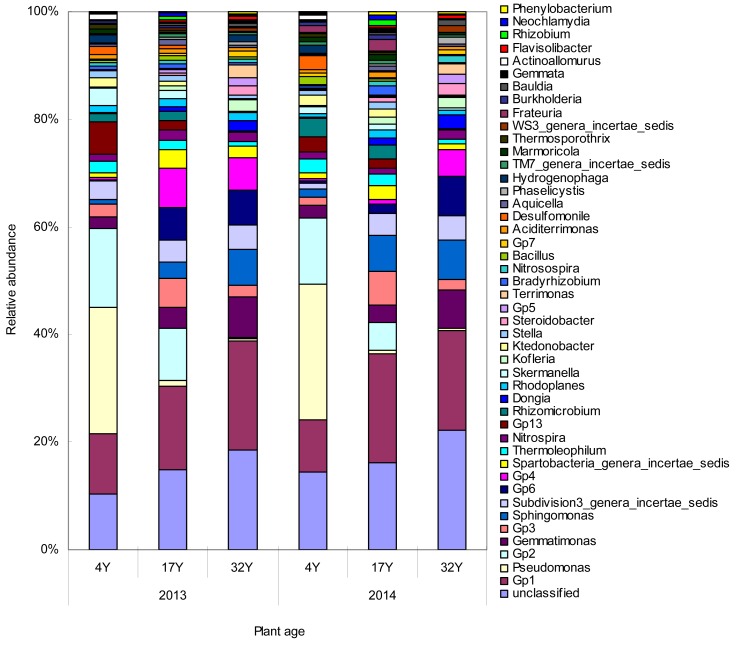
Relative abundance of genera in the nitrogen application of 4-year-old (4Y), 17-year-old (17Y), and 32-year-old (32Y) mulberry field soils. Relative abundances are based on the proportional frequencies of those DNA sequences that could be classified.

At the genus level, bacterial community (45 genera, relative abundance ratio of genera greater than 0.3%) mainly included *Pseudomonas*, *Gemmatimonas*, *Sphingomonas*, *Thermoleophilum*, *Nitrospira*, *Rhizomicrobium* and GP 1–5 sp. etc. (Fig 3). Of the 18 genera that were significantly affected by the plant age, seven belong to *Acidobacteria* (S5 Table). Of the 19 genera that were different (*P* < 0.05) between the 4Y and the 32Y soils, seven belong to *Proteobacteria* and eight belong to *Acidobacteria* (Table 3). The dominant genera in soils with different nitrogen application ages were different (Fig 3, Table 4). *Pseudomonas* was the primary dominant bacteria in the 4Y soil, accounting for 23.5% and 25.2% of the total bacteria in 2013 and 2014, respectively. However, in the 17Y and 32Y soils, *Pseudomonas* accounted for less than 1.16% of the total bacteria, which showed that the relative abundance of *Pseudomonas* significantly decreased as the nitrogen application ages increased (*P* < 0.05; Table 4) and significantly positively correlated with the pH (r = 0.699, *P* < 0.05) and soil organic matter content (r = 0.736, *P* < 0.01) (Table 5). However, the relative abundances of Gp1, Gp4 and Gp6 in the 4Y soil were significantly lower than that of in the 17Y and 32Y soils (*P* < 0.05; Table 4) and negatively correlated with the pH and soil organic matter content (Table 5).

**Table 3:** Comparisons (T-test) for genus abundance between the nitrogen application of 4-year-old (4Y), 17-year-old (17Y), and 32-year-old (32Y) mulberry field soils.

**Phyla**	**genus**	**Relative fold change**	*P* value (* *P* <0.05, ** *P* <0.01)
		**4 years / 17 years**	
*Proteobacteria*	*Pseudomonas*	16.34	0.000**
	*Rhizomicrobium*	9.52	0.003**
	*Sphingomonas*	-3.21	0.004**
	*Hydrogenophaga*	8.64	0.004**
	*Kofleria*	-3.13	0.005**
	*Stella*	-1.64	0.024*
	*Rhizobium*	-5.31	0.038*
*Acidobacteria*	*Gp4*	-11.28	0.000**
	*Gp1*	-1.38	0.001**
	*Gp6*	-10.26	0.001**
	*Gp3*	-4.24	0.002**
	*Gp13*	9.67	0.003**
	*Gp5*	-4.13	0.007**
*Bacteroidetes*	*Terrimonas*	5.26	0.002**
*Gemmatimonadetes*	*Gemmatimonas*	2.35	0.004**
*Planctomycetes*	*Gemmata*	2.34	0.015*
*Nitrospira*	*Nitrospira*	-2.64	0.028*
*Firmicutes*	*Bacillus*	-4.94	0.047*
**Phyla**	**genus**	**Relative fold change**	*P* value (* *P* <0.05, ** *P* <0.01)
		**4 years / 32 years**	
*Proteobacteria*	*Pseudomonas*	18.17	0.000**
	*Rhizomicrobium*	10.23	0.003**
	*Frateuria*	16.02	0.001**
	*Burkholderia*	8.69	0.005**
	*Bradyrhizobium*	2.57	0.005**
	*Hydrogenophaga*	10.67	0.011*
	*Rhizobium*	1.24	0.024*
*Acidobacteria*	*Gp2*	9.52	0.000**
	*Gp4*	-12.97	0.000**
	*Gp1*	-1.43	0.000**
	*Gp6*	-11.69	0.000**
	*Gp13*	12.31	0.004**
	*Gp5*	-3.46	0.013*
	*Gp7*	-2.18	0.032*
	*Gp16*	-0.55	0.042*
*Bacteroidetes*	*Terrimonas*	-15.43	0.001**
*Gemmatimonadetes*	*Gemmatimonas*	-2.56	0.002**
*Chloroflexi*	*Ktedonobacter*	10.89	0.004**
*Firmicutes*	*Bacillus*	2.01	0.039*

**Table 4:** The relative abundance of genera (had significant differences) in the nitrogen application of 4-year-old (4Y), 17-year-old (17Y), and 32-year-old (32Y) mulberry field soils in 2013 and 2014.

Genera	2013			2014		
	4Y	17Y	32Y	4Y	17Y	32Y
*Gp1*	4725±66.8^c^	10280±145.2^a^	8580±403.7^b^	4163±435.3^b^	9025±422.9^a^	8263±251.3^a^
*Pseudomonas*	9906±338.9^a^	769±94.5^b^	184±39.3^c^	10746±356.2^a^	276±56.9^b^	184±45.2^b^
*Gp2*	6203±391.0^a^	6372±123.3^a^	56±18.6^b^	5207±125.3^a^	2346±113.8^b^	33±10.1^c^
*Gemmatimonas*	930±124.2^b^	2546±254.8^a^	3184±214.3^a^	1046±98.6^b^	1354±104.6^b^	3135±219.5^a^
*Gp3*	976±66.9^b^	3680±201.3^a^	907±98.9^b^	682±89.5^b^	2857±187.5^a^	936±102.3^b^
*Sphingomonas*	364±31.5^c^	1928±154.2^b^	2878±218.2^a^	556±61.1^b^	2964±157.2^a^	3175±194.6^a^
*Gp6*	151±12.1^c^	4050±216.2^a^	2744±184.6^b^	243±57.1^c^	703±102.4^b^	3316±142.3^a^
*Gp4*	117±31.8^c^	4914±166.8^a^	2574±135.4^b^	152±25.9^b^	401±34.5^b^	2203±146.5^a^
*Thermoleophilum*	992±123.5^a^	1163±140.1^a^	369±53.1^b^	1047±108.9^a^	953±97.8^a^	322±30.1^b^
*Gp13*	2553±149.8^a^	1113±78.2^b^	22±7.8^c^	1260±98.4^a^	728±54.8^b^	28±6.7^c^
*Rhizomicrobium*	584±42.8^b^	1258±88.1^a^	119±10.2^c^	1443±123.5^a^	1194±105.4^a^	95±10.9^b^
*Dongia*	140±10.7^b^	575±49.4^b^	821±84.8^a^	113±9.2^c^	564±39.3^b^	1151±87.5^a^
*Skermanella*	1395±120.4^a^	1067±127.5^a^	93±9.7^b^	524±41.2^a^	550±50.4^a^	136±9.5^b^
*Gp5*	30±6.4^c^	363±30.2^b^	671±45.1^a^	38±13.8^b^	123±14.3^b^	815±89.0^a^
*Terrimonas*	39±11.5^b^	237±13.1^b^	1032±204.6^a^	53±19.8^b^	61±21.1^b^	825±67.8^a^
*Bacillus*	227±39.7^b^	558±36.5^a^	129±22.6^c^	614±46.8^a^	167±30.1^b^	136±28.8^b^
*Desulfomonile*	657±44.1^a^	443±35.2^b^	15±5.9^c^	1056±123.4^a^	145±20.8^b^	43±7.8^b^
*Hydrogenophaga*	638±19.5 ^a^	19±6.7^c^	494±83.1^b^	699±45.5^a^	18±5.2^b^	11±4.9^b^

The data are expressed as the means ± SD (*n* = 3). The superscript letters that differ within a column indicate significant differences between treatments (*P* < 0.05).

**Table 5:** Correlation analysis of the genera relative abundances with the physicochemical parameters (n = 18).

Genera	Pearson’s correlation coefficient				
	pH	SOM	Available N	Available P	Available K
*Pseudomonas*	0.699*	0.736**	0.622*	0.261	0.426
*Gemmatimonas*	-0.659*	-0.442	-0.273	-0.446	-0.452
*Sphingomonas*	-0.710**	-0.690*	-0.448	-0.250	-0.391
*Thermoleophilum*	0.473	0.292	0.053	0.758**	0.786**
*Nitrospira*	-0.169	-0.268	-0.302	0.135	0.006
Gp1	-0.382	-0.721**	-0.706**	0.012	-0.164
Gp2	0.596*	0.339	0.230	0.627**	0.670*
Gp3	-0.177	-0.388	-0.437	0.404	0.359
Gp6	-0.491	-0.455	-0.351	-0.277	-0.227
Gp4	-0.353	-0.382	-0.418	-0.014	-0.142
Gp13	0.720**	0.398	0.192	0.446	0.315
*Rhizomicrobium*	0.260	0.045	0.054	0.637*	0.673*
*Dongia*	-0.642*	-0.419	-0.238	-0.440	-0.591
*Rhodoplanes*	-0.115	-0.327	-0.663*	0.294	0.166
Skermanella	0.450	0.337	0.072	0.480	0.475

* significant at *P* < 0.05

** significant at *P* < 0.01

## Discussion

### Effects on bacterial community composition

The dominant phyla *Proteobacteria* and *Acidobacteria* counted for more than 70% of the bacterial abundance, which is nearly two times higher than the abundances of those phyla reported by Lauber et al. [23] and Chu et al. [24]. In addition, the *Acidobacteria* in the 17Y and 32Y soils were nearly two times higher than that of in the 4Y soil (Fig 2), and most of genera that significantly affected by the nitrogen application belong to *Acidobacteria* (S4 Table). This result may be own to the soil organic matter and pH, especially for the pH (The pH in the 32Y and 17Y soils were significantly lower than those found in the 4Y soil because of the long-term application of inorganic nitrogen fertilizer) (S1 Table). Soil pH has been recently documented in various soils as the major factor in determining the soil bacterial community composition and diversity. For example, soil pH has been shown to influence bacterial communities in soils across North and South America [23], British soils [25], and Changbai Mountain soils [26]. The effects of soil pH on the relative abundance of *Acidobacteria* in this study are consistent with these studies, which indicated that the relative abundance of *Acidobacteria* has often been observed to increase toward lower pHs [23, 24, 26–28]. Thus, our results further emphasize that soil pH plays an important role in shifting the bacterial community composition under the long-term application of nitrogen fertilizer.

### Effects on bacterial diversity

Previous reports of nitrogen fertilizer on the soil bacterial diversity were different [5, 8, 10, 29]. For example, Luo et al. [8] reported that long-term mineral nitrogen fertilizer treatment resulted in lower soil bacterial diversity in a maize-maize-soybean rotation in northeast China. However, Lupwayi et al. [10] and Ogilvie et al. [5] suggested that the application of a certain amount of nitrogen had no effect on the soil bacterial diversity. While, Yu et al. [16] reported that the long-term (16 years) application of inorganic nitrogen fertilizer at 450 kg N ha^–1^ year^–1^ significantly decreased the bacterial diversity compared with organic-inorganic fertilizer treatments. The results of this study also suggested that bacterial diversity in response to 17- and 32-year-old mulberry under the application of inorganic nitrogen fertilizer decreased, especially for the 32-year-old mulberry (Table 1). Therefore, the response of soil bacteria to nitrogen fertilizers depends on the fertilizer rate and duration of application. However, for mulberry, long-term application of inorganic nitrogen fertilizer could reduce the soil bacterial diversity.

### Effects of planting ages

Previous reports studying tea, cucumber, tomato, cotton and tobacco soils all indicated that soil microbial activity and diversity were significantly reduced as the age of the plants increased [30–32]. Fu et al. [33] also argued that the microbial communities in soils planted with asparagus decreased substantially with increasing plant age. However, in our opinion, the reduction in microbial diversity of 17- and 32-year-old soils did not completely contribute to the plant age but was closely related to fertilizer management. This result was confirmed in our previous study [16], which indicated that the microbial diversity in soils with long-term (16 years) application of organic-inorganic fertilizer treatment were significantly higher than that of in soils with long-term (16 years) application of nitrogen fertilizer treatment. Thus, the mechanisms by which different plants respond to plant age or fertilizers require further research.

## Conclusions

Long-term application of inorganic nitrogen fertilizer significantly altered the soil bacterial community composition and diversity. The soil bacterial relative abundance and diversity index generally decreased when inorganic nitrogen fertilizers were applied for 32 years. The relative abundance of *Pseudomonas* significantly decreased as the nitrogen application ages increased and positively correlated with the pH and soil organic matter content, whereas the relative abundances of *Acidobacteria* Gp 1, Gp4 and Gp6 significantly increased and negatively correlated with the pH and soil organic matter content. The stepwise regression analysis also indicated that OTUs relative abundance and Shannon index of bacteria were mainly correlated with the pH and soil organic matter content. Therefore, future research should focus on the intrinsic mechanisms underlying changes in soil microbial diversity, based on soil quality.

## Supporting Information

S1 Table The physicochemical properties of the 4-year-old (4Y), 17-year-old (17Y), and 32-year-old (32Y) mulberry field soils in November 2013 and 2014. *The data are expressed as the means ± SD (*n* = 3). The superscript letters that differ within a column indicate significant differences between treatments (*P* < 0.05). ^a^ Soil organic matter (SOM).
(DOC)

S2 Table Raw reads, sequences and OTUs of the 4-year-old (4Y), 17-year-old (17Y), and 32-year-old (32Y) mulberry field soils. (DOC)

S3 Table Variables correlated with the microbial properties or indicators obtained from the stepwise regression analysis in mulberry field soils with different plant ages. SOM: soil organic matter; ** significant at P < 0.01; *** significant at P < 0.001.
(DOC)

S4 Table Correlation analysis of the phyla relative abundances with the physicochemical parameters (N = 18). * significant at *P* < 0.05, ** significant at *P* < 0.01
(DOC)

S5 Table Results of one-factorial analyses of variance on differences of plant age. F- and *P*-values and associated degrees of freedom are listed.
(DOC)
