# Supplementary material for: Response of Bacteria Community to Long-Term Inorganic Nitrogen Application in Mulberry Field Soil
Source: PLoS One. 2016 Dec 15;11(12):e0168152. doi: 10.1371/journal.pone.0168152 (PMC5158035; doi:10.1371/journal.pone.0168152)
Supplement: S1 Table — *The data are expressed as the means ± SD (n = 3). The superscript letters that differ within a column indicate significant differences between treatments (P < 0.05). a Soil organic matter (SOM). (DOC) [file pone.0168152.s001.doc]

**Table S1 The physicochemical properties of the 4-year-old (4Y), 17-year-old (17Y), and 32-year-old (32Y) mulberry field soils in November 2013 and 2014.**

|  |  | pH(H_2_O) | SOM^a^ (%) | Available N (mg kg^–1^) | Available P (mg kg^–1^) | Available K (mg kg^–1^) |
| --- | --- | --- | --- | --- | --- | --- |
| 2013 | 4Y | 6.46±0.13^a^ | 2.58±0.02^a^ | 50.05±1.75^a^ | 44.36±1.02^ab^ | 126.50±2.50^a^ |
|  | 17Y | 5.65±0.11^b^ | 1.52±0.05^b^ | 35.00±2.53^b^ | 50.43±2.93^a^ | 131.50±5.50^a^ |
|  | 32Y | 5.06±0.09^c^ | 1.61±0.02^b^ | 37.25±3.12^b^ | 28.21±1.08^b^ | 101.00±3.00^b^ |
| 2014 | 4Y | 6.19±0.11^a^ | 2.22±0.08^a^ | 56.53±1.12^a^ | 41.59±1.02^ab^ | 125.50±4.00^a^ |
|  | 17Y | 5.59±0.10^b^ | 1.42±0.02^b^ | 36.40±1.09^c^ | 47.29±2.93^a^ | 128.00±5.00^a^ |
|  | 32Y | 5.45±0.06^b^ | 1.65±0.03^b^ | 47.95±0.92^b^ | 28.75±1.08^b^ | 95.00±2.50^b^ |

*The data are expressed as the means ± SD (*n* = 3). The superscript letters that differ within a column indicate significant differences between treatments (*P* < 0.05). ^a^ Soil organic matter (SOM).
