# Supplementary material for: Response of Bacteria Community to Long-Term Inorganic Nitrogen Application in Mulberry Field Soil
Source: PLoS One. 2016 Dec 15;11(12):e0168152. doi: 10.1371/journal.pone.0168152 (PMC5158035; doi:10.1371/journal.pone.0168152)
Supplement: S2 Table — (DOC) [file pone.0168152.s002.doc]

**Table S2** Raw reads, sequences and OTUs of the 4-year-old (4Y), 17-year-old (17Y), and 32-year-old (32Y) mulberry field soils.

| Group | Raw read | High quality sequence | OTUs |
| --- | --- | --- | --- |
| 4Y | 321344 | 306858 | 8523 |
| 17Y | 344612 | 329354 | 7067 |
| 32Y | 309190 | 295228 | 6844 |
