# Supplementary material for: Response of Bacteria Community to Long-Term Inorganic Nitrogen Application in Mulberry Field Soil
Source: PLoS One. 2016 Dec 15;11(12):e0168152. doi: 10.1371/journal.pone.0168152 (PMC5158035; doi:10.1371/journal.pone.0168152)
Supplement: S3 Table — SOM: soil organic matter; ** significant at P < 0.01; *** significant at P < 0.001. (DOC) [file pone.0168152.s003.doc]

**Table S3** Variables correlated with the microbial properties or indicators obtained from the stepwise regression analysis in mulberry field soils with different plant ages.

| Dependents | Variables related | R^2^ |
| --- | --- | --- |
| Shannon index of bacterial community | SOM^a^, pH | 0.582^**^ |
| OTUs relative abundance of bacteria | SOM^a^, pH | 0.523^***^ |

SOM: soil organic matter; ^**^ significant at P < 0.01；^***^ significant at P < 0.001.
