# Supplementary material for: Response of Bacteria Community to Long-Term Inorganic Nitrogen Application in Mulberry Field Soil
Source: PLoS One. 2016 Dec 15;11(12):e0168152. doi: 10.1371/journal.pone.0168152 (PMC5158035; doi:10.1371/journal.pone.0168152)
Supplement: S4 Table — * significant at P < 0.05, ** significant at P < 0.01 (DOC) [file pone.0168152.s004.doc]

**Table S4** Correlation analysis of the phyla relative abundances with the physicochemical parameters (n=18).

| Phyla | Pearson’s correlation coefficient | | | | |
| --- | --- | --- | --- | --- | --- |
|  | pH | SOM | Available N | Available P | Available K |
| *Proteobacteria* | 0.648* | 0.762** | 0.652* | 0.122 | 0.293 |
| *Acidobacteria* | -0.617* | -0.663* | -0.314 | 0.071 | -0.118 |
| *Verrucomicrobia* | 0.063 | -0.021 | -0.385 | 0.307 | 0.322 |
| *Gemmatimonadetes* | 0.554 | 0.534 | 0.313 | 0.307 | 0.373 |
| *Bacteroidetes* | 0.382 | 0.533 | 0.296 | 0.232 | 0.329 |
| *Actinobacteria* | -0.306 | -0.604* | -0.690 | -0.102 | -0.290 |

* significant at *P* < 0.05, ** significant at *P* < 0.01
