# Supplementary material for: Response of Bacteria Community to Long-Term Inorganic Nitrogen Application in Mulberry Field Soil
Source: PLoS One. 2016 Dec 15;11(12):e0168152. doi: 10.1371/journal.pone.0168152 (PMC5158035; doi:10.1371/journal.pone.0168152)
Supplement: S5 Table — F- and P-values and associated degrees of freedom are listed. (DOC) [file pone.0168152.s005.doc]

**Table S5** Results of one-factorial analyses of variance on differences of plant age. F- and *P*-values and associated degrees of freedom are listed.

|  | Bacterial genus | Degree of freedom | F value | *P* value |
| --- | --- | --- | --- | --- |
| *Acidobacteria* | *Gp1* | 3; 11 | 8.740 | 0.007 |
|  | *Gp2* | 3; 11 | 16.436 | 0.001 |
|  | *Gp3* | 3; 11 | 21.857 | 0.000 |
|  | *Gp6* | 3; 11 | 10.576 | 0.004 |
|  | *Gp13* | 3; 11 | 8.112 | 0.008 |
|  | *Gp5* | 3; 11 | 5.610 | 0.023 |
|  | *Gp4* | 3; 11 | 6.819 | 0.016 |
| *Proteobacteria* | *Pseudomonas* | 3; 11 | 5.622 | 0.022 |
|  | *Sphingomonas* | 3; 11 | 7.786 | 0.009 |
|  | *Rhizomicrobium* | 3; 11 | 8.586 | 0.007 |
|  | *Dongia* | 3; 11 | 5.026 | 0.031 |
|  | *Skermanella* | 3; 11 | 8.103 | 0.008 |
|  | *Desulfomonile* | 3; 11 | 5.256 | 0.026 |
|  | *Hydrogenophaga* | 3; 11 | 5.598 | 0.023 |
| *Gemmatimonadetes* | *Gemmatimonas* | 3; 11 | 15.374 | 0.001 |
| *Actinobacteria* | *Thermoleophilum* | 3; 11 | 4.456 | 0.040 |
| *Bacteroidetes* | *Terrimonas* | 3; 11 | 9.254 | 0.006 |
| *Firmicutes* | *Bacillus* | 3; 11 | 4.451 | 0.040 |
